# Improving the Stability and Effectiveness of Immunotropic Squalene Nanoemulsion by Adding Turpentine Oil

**DOI:** 10.3390/biom13071053

**Published:** 2023-06-29

**Authors:** Olga A. Krasnova, Vladislav V. Minaychev, Vladimir S. Akatov, Roman S. Fadeev, Anatoly S. Senotov, Margarita I. Kobyakova, Yana V. Lomovskaya, Alexey I. Lomovskiy, Alyona I. Zvyagina, Kirill S. Krasnov, Yuriy V. Shatalin, Nikita V. Penkov, Vitaly K. Zhalimov, Maxim V. Molchanov, Yuliya A. Palikova, Arkady N. Murashev, Eugeny I. Maevsky, Irina S. Fadeeva

**Affiliations:** 1 Institute of Theoretical and Experimental Biophysics, Russian Academy of Sciences, Pushchino 142290, Russia; okras.iteb@gmail.com (O.A.K.); vminaychev@gmail.com (V.V.M.); vladimir.akatov@gmail.com (V.S.A.); fadeevrs@gmail.com (R.S.F.); a.s.senotov@gmail.com (A.S.S.); kobyakovami@gmail.com (M.I.K.); yannalomovskaya@gmail.com (Y.V.L.); lomovskyalex@gmail.com (A.I.L.); alennazvyagina@gmail.com (A.I.Z.); kirill.krasnov64@gmail.com (K.S.K.); yury.shatalin@yandex.ru (Y.V.S.); vitaly.zhalimov@gmail.com (V.K.Z.); lvlaks.m@gmail.com (M.V.M.); maevsky.eugene@gmail.com (E.I.M.); 2 Pushchino State Institute of Natural Science, Pushchino 142290, Russia; murashev@bibch.ru; 3 Institute of Cell Biophysics RAS, Federal Research Center “Pushchino Scientific Center for Biological Research of the Russian Academy of Sciences”, Pushchino 142290, Russia; nvpenkov@rambler.ru; 4 Branch of Shemyakin-Ovchinnikov Institute of Bioorganic Chemistry, Russian Academy of Sciences, Pushchino 142290, Russia; yuliyapalikova@bibch.ru

**Keywords:** turpentine oil, squalene nanoemulsion, improving emulsion stability, immunopotentiating properties, bioactive terpenes

## Abstract

Turpentine oil, owing to the presence of 7–50 terpenes, has analgesic, anti-inflammatory, immunomodulatory, antibacterial, anticoagulant, antioxidant, and antitumor properties, which are important for medical emulsion preparation. The addition of turpentine oil to squalene emulsions can increase their effectiveness, thereby reducing the concentration of expensive and possibly deficient squalene, and increasing its stability and shelf life. In this study, squalene emulsions were obtained by adding various concentrations of turpentine oil via high-pressure homogenization, and the safety and effectiveness of the obtained emulsions were studied in vitro and in vivo. All emulsions showed high safety profiles, regardless of the concentration of turpentine oil used. However, these emulsions exhibited dose-dependent effects in terms of both efficiency and storage stability, and the squalene emulsion with 1.0% turpentine oil had the most pronounced adjuvant and cytokine-stimulating activity as well as the most pronounced stability indicators when stored at room temperature. Thus, it can be concluded that the squalene emulsion with 1% turpentine oil is a stable, monomodal, and reliably safe ultradispersed emulsion and may have pleiotropic effects with pronounced immunopotentiating properties.

## 1. Introduction

Owing to their structural features and polypharmacological properties, natural compounds are still the main sources of drug discovery [1]. Isoprenoids are among the most promising classes of chemical compounds for drug development [2,3].

The natural triterpene squalene is among the most popular terpenes in the pharmaceutical industry owing to its wide range of therapeutically significant biological properties [4]. Squalene is a normal metabolite in the animal body, representing an intermediate in the synthesis of cholesterol, which is part of biological membranes. Similarly, it is a precursor of phytosterol and ergosterol in plants and fungi [5]. In addition to its natural origin [6], in vitro and in vivo studies have shown the pronounced antioxidant properties of squalene because of its ability to reduce the concentrations of stress-induced ROS in cells [7], as well as its anti-inflammatory activity, which suppresses the expression of proinflammatory genes in immune cells and reduces the secretion of pro-inflammatory cytokines. Owing to these characteristics, squalene has become a “reference” carrier of drugs, including antitumor drugs such as squalene-gemcitabine conjugates and squalene-adenosine-α-tocopherol nanoparticles, aimed at enhancing the delivery of chemotherapy and removing the hyperinflammatory immune response [8,9]. Its emollient and UV-protective properties make it possible to actively include squalene and its saturated form, squalane, in the composition of cosmetic and cosmeceutical agents [6], but squalene has found the most widespread use as the main component of emulsion adjuvants MF59 (Novartis), AS03 (GlaxoSmithKline, GSK), and AF03 (Sanofi) in influenza virus vaccines [10].

First-generation emulsion adjuvants use mineral oils, which manifest themselves as strong potentiators of the humoral immune response; however, they are not metabolized by the body and cause the formation of aseptic abscesses, which does not allow for the use of such substances for human vaccination [11,12]. The development of oil-in-water emulsions based on fully metabolizable components, primarily squalene, has made it possible to obtain improved seasonal inactivated influenza vaccines approved in Europe and the USA, followed by vaccines against avian influenza (H5N1 and other strains) and pandemic influenza (H1N1) [13,14]. Currently, the generally recognized high safety profile of squalene emulsions allows them to be used for the vaccination of a wide range of people, such as adults, the elderly, children, infants, and pregnant women [15].

The highest demand for squalene in the next few years is expected to remain in the pharmaceutical vaccine market, and the total value of the global squalene market will increase to USD 184 million by 2025 [16]. The global demand for squalene is increasing against the background of the SARS-CoV-2 pandemic and the urgent need for immunization of all population groups. However, this creates the risk that the existing volume of squalene produced will soon become insufficient and will entail a shortage of vaccine doses and hence the impossibility of prompt vaccination [4].

The largest natural source of squalene is fat secreted from the liver of deep-sea sharks [17]; however, intensive fishing of squalene threatens the existence of these populations [16]. Currently, the production of squalene from plants, in particular from olive oil, has been established; however, phytosqualene accounts for only half of the world market, which does not compensate for the volume of squalene obtained from sharks and creates the need to develop more cost-effective technologies for its production [18]. One of the leading directions in this field is the microbiological production of squalene by cellular and genetic engineering of bacteria and yeast that accumulate squalene [19,20,21,22]. However, mass application of this technology requires a large amount of money and time. As a result, the search for alternative ways to prevent and eliminate squalene deficiency remains relevant; namely, reducing its dosage in preparations without losing its effectiveness by modifying the composition or increasing the stability of squalene emulsions, which allows for the production of large batches at the same time and reduces production costs.

Squalene is the main, but not the only, isoprenoid used as a vaccine adjuvant. It is also known from the literature that at the early stages of vaccination development, turpentine oil (obtained from pine sap) was also used as an adjuvant [23]; however, with bolus administration in its pure form, the development of abscesses, necrosis, and pronounced painful sensations at the injection site were observed; therefore, its use was discontinued. However, a number of studies have indicated that in an emulsified form, turpentine oil can exert an adjuvant effect more effectively and safely during vaccination [24,25,26,27,28].

Turpentine oil is a mixture of isoprenoids obtained by distilling the volatile fraction of pine sap (resin released when wood is damaged). Turpentine contains 9–50 different molecules, including bicyclic monoterpenes, diterpenes, polyprenols, terpe-nacetates, and aromatic compounds. Turpentine oil is a mixture listed by the European Chemical Agency (ECHA) under CAS number [8006-64-2] and contains mostly (up to 89%) bicyclic monoterpenes such as α-pinene, β-pinene, 3-karen, camphene, *cis*-pinane, dihydropinene, and monocyclic terpenes such as limonene, terpinene, terpinolene, and fellandren [29,30]. According to GOST 1571-82, in the composition of turpentine oil according to OKP 24 1611 0120 (top grade), the mass fraction of the sum of α-pinene and β-pinene is at least (and on average) 60%, as a result of which a significant therapeutic effect in turpentine oil is possible for pinenes and conditionally significant for δ-3-karen and α-longifolene, the mass fraction of which can reach 10% [31,32].

α-Pinene is a bicyclic hydrocarbon of two isoprene units with the general formula C_10_H_16_. The biological activity of α-pinene has been sufficiently studied and its effects on a wide range of biological processes have been reliably established. Among the most significant, there is a pronounced antibacterial effect against several strains of Gram-negative and Gram-positive bacteria, including against methylline-resistant *Staphylococcus aureus* (IC50 = 68.6 ± 7.9 μg/mL) and *E.coli* [33,34]. In addition, the antifungal effect of α-pinene on yeast of the genus Candida exceeded the effectiveness of the reference fungicide clotrimazole [35]. In the context of the immunotropic properties of α-pinene, it is worth noting that its anti-inflammatory activity involves suppressing the expression of the main inflammatory mediators, such as nuclear factor NF-kB, tumor necrosis factor-α (TNF-α), and interleukin-6 (IL-6), as well as additional inflammatory factors, such as mitogen-activated protein kinases (MAPK), inducible nitric oxide synthase (iNOS), and cyclooxygenase-2 (COX-2) [36,37]. Moreover, α-pinene prevented apoptotic cell death induced by UVA- and H_2_O_2_-mediated oxidative stress by suppressing the expression of apoptotic Bax/Bcl-2 genes and, accordingly, deactivating the caspase cascade owing to its antioxidant properties, probably because of the chemical structure of the compound [38,39]. However, in transformed cells, for example, in human ovarian cancer cells, high concentrations of α-pinene (approximately 100 μg/mL) cause a polar effect: stimulation of apoptosis against the background of an increase in the concentration of caspase-3, which makes it possible to use α-pinene as a starting point for the development of antitumor drugs [40].

Studies on the enantioselective properties of α-pinene have shown that (+)-α-pinene exhibits more pronounced antibacterial, antifungal, antimalarial, and anti-inflammatory activities than the negative form [41,42,43], while the antibacterial properties are due to the ability of (+)-α-pinene to inhibit the activity of phospho-lipase and esterase in bacteria [44]. In contrast to the positive enantiomer, (−)-α-pinene demonstrated the ability to inhibit the infectious bronchitis virus (IC_50_ 0.98 ± 0.25 mM) [45]. The properties of both enantiomers are extremely valuable to the pharmaceutical industry, indicating their great potential for use in complexes.

Surprisingly, despite isomerism, the properties of α- and β-pinenes are different. As for the α-isomer, antimicrobial, anticancer, anti-inflammatory, antiallergic, and antioxidant properties have been shown for β-pinene [46]; however, their degree of severity is not the same, although there is a tendency for greater therapeutic activity in (+)-enantiomers. Thus, (+)-β-pinene has approximately 2–12 times higher antimicrobial activity than (+)-α-pinene, both against Gram-positive and Gram-negative bacteria (*E. coli*, *S. aureus* and *Bacillus cereus*), as well as *Candida albicans* yeast [47,48]. This may be due to the fact that the lipophilic β-pinene damages the cellular integrity of microorganisms more than the α-isomer. In contrast, antimalarial activity is more pronounced in α-pinene, exceeding the parameters of the β-isomer by 250 times [47]. A similar situation is observed in terms of anti-inflammatory properties; the β-isomer slightly suppresses inflammatory processes and is a weak inhibitor of neutrophil chemotaxis. The target of β-pinene is assumed to be the transient receptor potential (TRP) channels (except TRPV1, TRPV3, and TRPM8). β-pinene, due to its lipophilicity, reacts with the hydrophobic pocket of TRP and thus regulates the opening and closing of the channel responsible for the mobilization of calcium ions in neutrophils, which leads to cell de-sensitization [49].

δ-3-Karen, which can contribute to the therapeutic effect of turpentine oil, has shown effective inhibitory activity against Gram-positive Brochothrixt hermosphacta and Gram-negative Pseudomonas fluorescens in studies of biological properties. It is assumed that 3-karen can disrupt the normal morphology of the bacterial cell wall and membrane, leading to the leakage of macromolecular substances and inhibition of the activity of the intracellular cycle of tricarboxylic acids and enzymes associated with glycolysis, disrupting the synthesis and breakdown of ATP, and leading to metabolic dysfunction [50].

A number of antimicrobial and antimycotic properties have also been shown for longifolene, which is conditionally therapeutically significant in the composition of turpentine oil; however, its sufficiently pronounced anti-inflammatory activity is more valuable and slightly inferior to that of (+)-α-pinene [51]. In addition, the composition of turpentine oil includes camphene, which has an analgesic effect due to the activation and desensitization of epidermal nociceptors, which is significant for parenterally administered drugs [52].

This wide range of pharmacologically valuable components in the composition of natural mixture makes turpentine oil an object of interest for pharmacological research. Summarizing the above, it should be concluded that turpentine oil, due to the presence of a significant pinene component and other terpenes, has pronounced analgesic, anti-inflammatory, immunomodulatory, antibacterial, and antitumor properties, which is extremely important in the context of the production of emulsion preparations and anticoagulant and antioxidant properties. In view of this, the addition of turpentine oil to squalene emulsions can increase their effectiveness, thereby reducing the concentration of expensive and possibly deficient squalene and increasing its stability and shelf life.

In this study, a technique for obtaining squalene emulsions with the addition of various concentrations of turpentine via high-pressure homogenization with the addition of surfactants was proposed as the most effective method of emulsification, and the stability, efficacy, and safety of the obtained emulsions under in vitro and in vivo conditions were studied.

## 2. Materials and Methods

### 2.1. Reagents

pHrodo Green *E. coli*, PE anti-mouse/human Ki-67 antibodies, PE Rat IgG2b k Isotype Ctrl, and IgE Mouse ELISA Kit were purchased from Thermo Scientific (Waltham, MA, USA). Span^®^ 85 (S7135), TWEEN^®^ 80 (S7135), Deuterium oxide (151882), Culture media DMEM and RPMI 1640, L-glutamine, Pokeweed Mitogen (PWM), phytohemagglutinin (PHA), cytochalasin D, Methyl Methanesulfonate, Saponin, ELISA kits Mouse IL-2 ELISA Kit, TWEEN^®^ 20, Human Serum Albumin, and other chemicals were purchased from Sigma-Aldrich (St. Louis, MO, USA). Turpentine Oil (State Standard 1571-82, Top Grade RPC 24 1611 0120) was purchased from «IB Smirnova» (Moscow, Russia). Drabkin Method Kit was purchased from Agat-Med Ltd. (Moscow, Russia). EasySep™ Mouse Monocyte Isolation Kit was purchased from StemCell Technologies Inc. (Vancouver, BC, Canada). Mouse IFN-gamma Quantikine ELISA Kit was purchased from R&D Systems Inc. (Minneapolis, MN, USA). Mouse TNF alpha ELISA Kit, Mouse IgM ELISA Kit, and Abcam Histamine ELISA kit were purchased from Abcam (Cambridge, UK). Mouse IgG ELISA Kit was purchased from Cayman Chemical (Ann Arbor, MI, USA). All other reagents used were of analytical reagent quality.

### 2.2. Preparation Procedure and Characterization

#### 2.2.1. Preparation of the Emulsions

In the first stage, a basic pre-emulsion was obtained: squalene (4,5%) and turpentine oil (0.03%, 0.1%, 0.3%, and 1.0%) were mixed separately on a vibrating mixer and surfactants (Span^®^ 85, 0.5% and TWEEN^®^ 80, 0.5%) were added; then, a citrate buffer (pH 6.0) was introduced in several stages, bringing the mixture to homogeneity using an IKA Ultra-Turrax T25 (IKA Works Inc., Wilmington, NC, USA). In the second stage, the resulting emulsions were transferred to a high-pressure plunger homogenizer («Donor-3»; ITEB RAS, Pushchino, Russia) and subjected to extrusion grinding at a chamber pressure of 400 bar and t = 27 ± 1° for four cycles. The pressure in the chamber was then increased to 800 bar, and grinding was performed at 38 ± 1 °C for at least six repetitions. The extrusion was completed with a homogenization cycle at a pressure of 200 bar, and the emulsion was left for Ostwald maturation for 3 days at +4 °C. In the third stage, the final emulsions was filtered under aseptic conditions at a pressure not exceeding 900 bar through a capsule membrane filter PS-065/020-A-500 with pore size 0.65/0.2 μm (NPP Technofilter LLC., Vladimir, Russia).

An emulsion that did not contain turpentine oil (similar in composition to the squalene emulsion MF59^®^ adjuvant, Novartis [53,54]) was obtained in accordance with the US6299884B1 patent via the high-pressure homogenization method using a high-pressure plunger homogenizer (“Donor-3”, ITEB RAS, Pushchino, Russia).

#### 2.2.2. Analysis of Dispersed Phase

Analysis of the dispersed phase (fractional composition) of the emulsions was performed via the freeze-lyophilization method using a low-temperature freezer New Brunswick Premium U410 (New Brunswick, NJ, USA) and Freeze Dryer FreeZone (Labconco, Kansas City, MO, USA). To perform the test, variants with a volume of 1 mL were placed in cryoprobes (preweighed), frozen at −80 °C, and lyophilized for 24 h until the pressure in the lyophilizer chamber stabilized and the weight of the variants stopped changing. The emulsions were then reweighed, and the compositions of the oil and water components were evaluated.

#### 2.2.3. PH Measurements

The pH of the emulsions was determined using a FiveEasy F20-Std-Kit pH meter (MetlerToledo Int., Greifensee, Switzerland) with mandatory stirring using a magnetic stirrer MR Hei-Mix S (Heidolph Instr. GmbH, Schwabach, Germany), with a sample volume of at least 10 mL. The pH measurement for each sample was performed at least 3 times.

#### 2.2.4. Dynamic Viscosity

The dynamic viscosity of the emulsions was measured using an Sinusoidal vibration Viscometer SV-10 (A&D Company Limited, Tokyo, Japan). Systematic instrument error did not exceed 3%. All measurements were performed at an ambient temperature of 22 °C at least three times.

#### 2.2.5. Dynamic Light Scattering (DLS) and ζ Potential

The colloidal stability of the emulsions was examined using a Zetasizer Nano ZS Pro Ultra analyzer (Malvern Panalytical Ltd., Malvern, UK) at 22 °C. The instrument settings were optimized automatically using the Zetasizer Software 7.13 (Malvern Panalytical Ltd., Malvern, UK). The average values were obtained from three readings, and all the measurements were performed five times. For the ζ-potential study, immediately before the measurement, dilution of the emulsions was performed with citrate buffer, and the stability of the emulsions was evaluated via dilution with deionized water (Milli Q Advantage, Millipore, Burlington, MA, USA).

#### 2.2.6. Nuclear Magnetic Resonance (NMR)

For each sample, sequential mixing was performed using an Eppendorf tube; to 540 µL of the sample, 60 µL of a 4 mM TSP solution was added to the phosphate buffer (pH 7.2) in D_2_O. The samples (600 µL) were placed in NMR-ampoules with a diameter of 5 mm. One-dimensional (1D) and two-dimensional (2D) nuclear magnetic resonance spectra were recorded on a Bruker 600 AVANCE III NMR spectrometer (Bruker Bio-Spin, Reinstetten, Germany). All measurements were performed at 298 K. The pulse sequences used in the experiments were the standard from the Bruker pulse sequence library. To suppress the signal from the water protons, a pre-saturation method was used using 1D pulse sequence ZGPR. The operating frequency for the protons was 600 MHz, the free induction decay (FID) was recorded during aq = 3.42 s at 96 k points, the spectrum width was 24 m.d., and a 90° pulse of 12 µs was used. When obtaining the NMR spectra, the time between the scans was 10 s. Two-dimensional spectra of homonuclear (1H-1H) COZY spin–spin correlation (COSYGPPRQF sequence) for the samples were recorded over the entire region containing the signals. The delay time between the COSY pulses was 1 s and the data volume was approximately 2048/512 points. To achieve an acceptable signal-to-noise ratio for the 1D spectra, the number of accumulations was 128 and 2 for the 2D spectra. Calibration of chemical shifts was carried out using the TSP signal at 0.00 m.d., acting as an internal standard. Ethyl alcohol spectra were excluded from the analysis.

#### 2.2.7. Thiobarbituric Acid-Reactive Substance Assay

The thiobarbituric acid-reactive substance (TBARS) test was used to investigate secondary oxidative products, such as malonaldialdehyde (MDA). The production of TBARS was measured using TBA method [55,56]. Ferrous sulfate was used as a lipid peroxidation catalyzer. Briefly, 1 mM FeSO_4_ was added to the 500 μL samples of lecithin (10 mg/mL) or emulsions (0%, 0.03%, 0.1%, 0.3%, and 1.0% turpentine oil). The mixtures were incubated at 37 °C for 30 min and 24 h. Then, 0.50 mL of TBA (0.67%) dissolved in 2% orthophosphoric acid was added to 0.50 mL of the mixtures. The mixtures were incubated at 100 °C for 1 h. After cooling, the resulting colored adduct was extracted from the mixtures with 1.0 mL of *n*-butanol. The adduct was detected at 532 nm using a Cary 100 Scan spectrophotometer (Varian, Sydney, Australia). 1,1,3,3-Tetraethoxypropane was used as an MDA standard. The concentration of MDA in the samples was calculated using the equation of approximation of the calibration line (5–50 μM MDA).

#### 2.2.8. Emulsion Stability Measurement

Emulsion stability was evaluated in accordance with the standards for testing drugs for parenteral administration [57,58,59,60]. Briefly, the prepared emulsions were poured into a 50 mL bottle for glass observation, sealed, and left to stand at different temperatures for different periods of time (*n* = 5 for each emulsion). Room temperature of 25 ± 2 °C was used as the highest possible storage temperature. The standard recommended storage temperature was 5 ± 3 °C. This procedure was performed for 12 months for all emulsions and 24 months for the pure squalene emulsion and emulsion with 1% turpentine oil. The following steps were used to characterize the emulsion: for all emulsions, to assess stability, the quantitative characteristics described in Section 2.2.3, Section 2.2.4, Section 2.2.5 and Section 2.2.6 were measured; for pure squalene emulsion and emulsion with 1% turpentine oil, to assess stability, the characteristics described in Section 2.2.3, Section 2.2.4 and Section 2.2.5 were measured; and the obtained parameters were compared with the parameters obtained for freshly prepared emulsions.

### 2.3. In Vitro Studies

#### 2.3.1. Cell Cultures

NIH/3T3 mouse embryonic fibroblasts cell culture were obtained from the ATCC (Manassas, VA, USA).

Monocytes were isolated from the peripheral blood of ICR (CD-1) outbred mice using the EasySep™ Mouse Monocyte Isolation Kit (Stem Cell Technologies, Vancouver, BC, Canada), in accordance with the manufacturer’s recommendations.

Human erythrocytes were isolated from the peripheral blood samples of healthy volunteers according to the method described by Brosseron et al. [61].

Mouse mononuclear cells were isolated as described by Houthuys et al. [62].

#### 2.3.2. Cytotoxicity Assay

This study used an NIH/3T3 mouse embryonic fibroblast cell culture. Cells were seeded at a amount of 3.5 × 10^4^ per well of a 96-well plate in DMEM culture medium with the addition of 10% fetal bovine serum (FBS) at 37 °C and 5% CO_2_ in a CO_2_ incubator (Binder CB150, Binder GmbH, Tuttlingen, Germany). Emulsions with different turpentine oil contents were added to the cultures 24 h after cell seeding. Cytotoxicity was assessed based on the ratio of the number of living cells in the experimental and control (non-treated) cultures 24 h after emulsion addition. Cytotoxicity analysis was performed by staining dead cells with a fluorescent dye propidium iodide (1 μg/mL) and estimating the number of dead cells using a BD Accuri C6 flow cytometer (BD Bioscience, Franklin Lakes, NJ, USA).

#### 2.3.3. Genotoxicity Assay

This study used an NIH/3T3 mouse embryonic fibroblasts cell culture. Cells were seeded in Petri dishes containing 3 × 10^5^ cells in 2 mL of DMEM with the addition of 10% FBS at 37 °C and 5% CO_2_ in a CO_2_ incubator (Binder CB150, Binder GmbH, Germany). Emulsions with different turpentine oil contents were added to cultures 24 h after cell seeding and incubated for 4 h. DNA damage was determined via alkaline gel electrophoresis of individual cells using the DNA comet method [63]. Analysis of the amount of DNA in the “tail” of the comet was carried out using OpenComet v1 software 3.1 (CometBio, Singapore). At least 500 DNA comets were analyzed. As a positive control, NIH/3T3 cells were treated with 20 µg/mL methyl methanesulfonate for 4 h. As a negative control, NIH/3T3 cells cultured in emulsion-free medium were used.

#### 2.3.4. Hemolysis Assay

This study was carried out using the colorimetric method of quantitatively determining the hemoglobin content via the hemiglobincyanide method using the Drabkin Method Kit. Previously, a 2% suspension of human peripheral blood erythrocytes prepared in saline solution was incubated with the emulsions at 37 ± 2 °C for 4 h. A 10% saponin solution prepared in saline was used as the positive control for erythrocyte lysis. The hemolytic activity was detected using an Infinite F200 Microplate Reader (Tecan, Männedorf, Switzerland) at a wavelength of 540 nm.

#### 2.3.5. Phagocytosis Assay

Monocytes were cultured in RPMI-1640 medium supplemented with 10% FBS and 2 mM L-glutamine solution at 37 °C and 5% CO_2_ in a CO_2_ incubator (Binder CB150, Binder GmbH, Germany). The cells were seeded at a density of 5 × 10^3^ cells/well in 100 µL of full growth medium in 96-well plates. After 24 h after seeding, the emulsions were added to the cells and incubated for 18 h. The phagocytic activity of the cells was assessed using the values of the phagocytic number and phagocytic index after 2 h of incubation in a CO_2_ incubator in a growth medium with the addition of 1 mg/mL pHrodo Green *E. coli*. To control nonspecific staining, cells were incubated with 10 μg/mL cytochalasin D for 30 min in a CO_2_ incubator, then 1 mg/mL pHrodo Green *E. coli* was added, and the incubation was continued for another 2 h. Fluorescence was measured using a BD Accuri C6 flow cytometer (BD Bioscience, Franklin Lakes, NJ, USA). Phagocytic activity was characterized based on the percentage of fluorescent cells (phagocytic index) and the average fluorescence intensity per cell (phagocytic number) in fluorescent cells [64,65]. Monocytes cultured in emulsion-free medium were used as a negative control.

#### 2.3.6. Mitogen Stimulation Assays (MSAs) on Proliferation Mononuclear Cells

Mononuclear cells were cultured in RPMI-1640 medium supplemented with 10% FBS and 2 mM L-glutamine solution at 37 °C and 5% CO_2_ in a CO_2_ incubator (Binder CB150, Binder GmbH, Germany). The cells were seeded at 5 × 10^4^ cells/well in 100 µL of full growth medium in 96-well plates. After 24 h after seeding, the emulsions were added to the cells and incubated for 72 h. To induce proliferation, mononuclear cells were incubated with 15 μg/mL of phytohemagglutinin (PHA) and 15 μg/mL of pokeweed mitogen (PWM) for 72 h. The proliferative activity of lymphocytes was determined based on the evaluation of the expression of the Ki-67 nuclear antigen before and after the induction of polyclonal activation and incubation with emulsions. To analyze the expression of Ki-67, PE anti-mouse/human Ki-67 antibodies were used to control the nonspecific binding of antibodies, and cells were stained with PE Rat IgG2b k Isotype Ctrl. Fluorescence was measured using a BD Accuri C6 flow cytometer (BD Bioscience, Franklin Lakes, NJ, USA). At least 30,000 cells were analyzed for each group.

### 2.4. In Vivo Studies

#### 2.4.1. Animals

The study was conducted at the Biological Testing Laboratory of the BIBCh RAS, accredited by the Association for Assessment and Accreditation of Laboratory Animal Care International (AAALAC), in accordance with the standards of the Guidelines for the Care and Use of Laboratory Animals (8th ed., Institute of Laboratory Animal Research). Outbred SPF male Wistar rats aged 7–8 weeks (190–215 g) at the beginning of the experimental procedures were obtained from the Nursery of Laboratory animals “Pushchino” in the Branch of Shemyakin-Ovchinnikov Institute of Bioorganic Chemistry Russian Academy of Sciences (BIBCh RAS), Russian Academy of Sciences (Pushchino). All animal manipulations complied with the International Principles of the World Health Organization Guidelines on Biomedical Research involving Animals and were reviewed and approved by the Institute’s Commission on Humane Treatment of Animals (IACUC Protocol number: 775/20, approval date: 14 December 2020). The animals were acclimatized for two weeks before the experimental procedures and kept in barrier rooms in a controlled environment: temperature 20–24 °C, relative humidity 30–60%, and 12 h lighting cycle. The animals received free access to feed (Mucedola Standard Diet 4RF21, Settimo Milanese, Italy) and Milli-RO prepared filtered tap water (Milli-Q HR 7060 High-Flow RO Water Purification System; Merck KGaA, Darmstadt, Germany). Dust-free rodent bedding consisting of wood chips (LIGNOCEL BK8/15; JRS, Rosenberg, Germany) was used. Animal cages were supplied with materials to enrich the environment: yurt-houses made of red transparent polycarbonate (Techniplast S.p.A, Buguggiate, Italy).

#### 2.4.2. Cytokine Measurement by ELISA

Changes in the levels of interleukin-2 (IL-2), interferon gamma (IFN-γ), and tumor necrosis factor alpha (TNF-α) in the blood serum obtained 72 h after subcutaneous administration of an experimental emulsions to outbred ICR (CD-1) mice were analyzed. Quantitative assessment of cytokines was carried out using commercial ELISA kits: Mouse IL-2 ELISA Kit, Mouse IFN-gamma Quantikine ELISA Kit, and Mouse TNF alpha ELISA Kit. The enzyme immunoassay was determined at 450 nm using an iMark plate reader (Bio-Rad, Hercules, CA, USA).

#### 2.4.3. Immunoglobulin Production Assay by ELISA

Assessment of the effect of emulsions on IgG, IgM, and IgE production was carried out based on the analysis of changes in the amount of immunoglobulins in peripheral blood serum obtained 24 h after subcutaneous administration of an experimental emulsions to outbred ICR (CD-1) mice. Quantitative evaluation of IgG, IgM, and IgE by enzyme immunoassay was carried out using commercial ELISA kits (Mouse IgG ELISA Kit, Mouse IgM ELISA Kit, and IgE Mouse ELISA Kit) and was determined at 450 nm using an iMark plate reader (Bio-Rad, Hercules, CA, USA).

#### 2.4.4. Histamine Production Assay by ELISA

Assessment of the effect of emulsions on histamine production was carried out based on the analysis of changes in the amount of histamine in peripheral blood serum obtained 24 h after subcutaneous administration of emulsions to ICR (CD-1) outbred mice. Quantitative evaluation of histamine by enzyme immunoassay was carried out using the Abcam Histamine ELISA kit and was determined at 450 nm using an iMark plate reader (Bio-Rad, Hercules, CA, USA).

#### 2.4.5. Maximum Injectable Dose (MID) Studies

The in vivo biocompatibility and safety of the emulsion were evaluated by determining the maximum injectable dose (MID) in outbred ICR (CD-1) mice in the Laboratory of Biological Testing (LBI) of the BIBCH RAS. The LBI BIBCH RAS is included in the Russian National GLP Compliance Program and has accreditation for compliance with the Principles of Good Laboratory Practice (Statement of OECD GLP Compliance No. G-044 dated 17 July 2021 (valid until 17 July 2023, Slovak National Accreditation Service). Animal procedures were reviewed and approved by the Bioethical Commission of the BIBCH RAS. All procedures were performed according to approved Standard Operating Procedures (SOP). No deviations affecting the quality, integrity, or interpretation of data were recorded.

This study included outbred male ICR (CD-1) mice. An emulsion with a maximal concentration of turpentine oil (1% turpentine oil only) was injected fractionally during the day via a subcutaneous injection at a standard volume of 10 mL/kg. The maximum allowable volume of subcutaneous injection for mice was 50 mL/kg; therefore, all animals received five injections of the test substance (or saline for control mice). After the administration of the substances, all animals were monitored for 48 h, their body weight and feed intake were recorded daily, and clinical signs of health abnormalities were assessed. At the end of the study (day 3), all the animals were euthanized using the carbon dioxide protocol. During necropsy, the external state of the body, internal organs, and tissues was examined.

#### 2.4.6. Xenospecific Antibody Measurement Using ELISA

This study was conducted using male ICR (CD-1) outbred mice. Immunization of laboratory animals with antigen (Human Serum Albumin, HAS HSA) in the amount of 50 µg of antigen per animal with or without the addition of 50 µL of emulsion was performed via double intraperitoneal administration of the suspension at an interval of 14 days. Venous blood samples were collected from laboratory animals 21 d after the first immunization, followed by serum production. To determine the titers of antigen-specific antibodies in the mouse sera, 100 µL of antigen at a concentration of 3 µg/mL was injected into the wells of a 96-well plate and incubated overnight at 4 °C. The plates were washed thrice with PBS containing 0.05% Tween 20 and incubated in 5% milk in PBS with 0.05% Tween 20 for 2 h at 22 °C. To determine the final titer of the antibodies, the sera were titrated at a multiplicity of 2 and incubated in the wells of tablets for 2 h + 37 °C. Mouse antibodies bound to the solid phase were detected using the standard peroxidase-antiperoxidase PAP method. Bound peroxidase in the wells was detected using an OPD, and the optical density of the wells was determined using an Infinite F200 Microplate Reader (Tecan, Männedorf, Switzerland) at a wavelength of 495 nm. The titer was calculated as the dilution that resulted in an OD495 of ≥0.3.

### 2.5. Statistical Analysis

In vitro results are presented as the mean ± standard deviation (M ± SD). Each in vitro experiment was performed at least five times (*n* ≥ 5). Statistical significance of the differences was determined using one-way ANOVA, followed by multiple Holm–Sidak comparisons (*p* < 0.05). The design of the experiment and related statistics (ANOVA) were performed using SigmaPlot™ 14.0 (Systat Software Inc., San Jose, CA, USA). Plots were created using SigmaPlot™ 14.0.

In vivo results are presented as mean ± standard deviation (M ± SD). Each experiment was performed at least thrice (*n* ≥ 3). Statistical significance of differences was determined using the Mann–Whitney U test (*p* < 0.05). NIH/3T3 mouse embryonic fibroblast cell cultures were obtained from the ATCC (Manassas, VA, USA).

## 3. Results

### 3.1. Results of Preparation and Physicochemical Characterization of Emulsions

The resulting series of emulsions with different turpentine oil contents was a homogeneous milky-white liquid without shade. Table 1 shows that the physicochemical characteristics of the emulsions did not differ regardless of the turpentine oil concentration. The fractional composition of the emulsion samples with different turpentine oil contents was determined as follows: 94–95%—aqueous fraction, 4.5 to 5.5%—oil fraction (terpenoid oils), and 1% surfactants (surfactants). The pH for samples of different compositions did not differ significantly, with an average value of 5.98 ± 0.01, which is equal to the pH of the citrate buffer used for the preparation of emulsions.

Simultaneously, the dynamic viscosity indices of the samples differed, which may be due to both the difference in the composition of the emulsions and the difference in the concentration of the dispersed phase. However, in total, the samples of all the presented formulations had stable viscosity, and the measurement error was *p* ≥ 0.01. The average viscosity value was 1180 ± 0.007 cPs, did not significantly differ among all the emulsions, and was commensurate with the viscosity of the water and buffer solution used for the preparation of emulsions (1002 cPs). The rheological characteristics of emulsions close to the water parameters are optimal and give the developed substance an advantage over more viscous emulsion preparations for parenteral administration, in terms of simplicity and ease of use [66,67,68].

In addition, the emulsions were studied before and after cut-off filtration by analyzing their granulometric characteristics, polydispersity index, and ζ-potential. The use of cut-off filtration in the technological process of emulsion preparation can be excluded because all emulsions are highly stable and homogeneous [54,69].

The particle size did not differ significantly among the groups; the average was 175.76 ± 51.7 nm, which is many times less than the threshold value for intravascular drugs (5 microns) and optimal for effective interaction with immune cells [59,70]. The particle size distribution for all samples was narrow-peaked, which indicates the unimodality of the resulting emulsions and was confirmed via the low values of the polydispersity index, which was 0.27 ± 0.02. The ζ-potential of the emulsions was equal to −3.09 ± 0.95 mV and did not significantly differ for the studied emulsions.

When measuring the concentration of TBARS (Table 2) in the emulsions, it was found that the concentration of the oxidized products increased with increasing incubation time with Fe(II) and turpentine oil. However, the obtained values did not exceed those corresponding to the standard lecithin emulsion. In turn, when comparing the results of t30 min and t24 h incubation, it can be seen that in all samples containing turpentine oil, the TBARS content did not significantly differ; however, in the pure squalene emulsion (without turpentine), the TBARS content increased by 2 times. The obtained data indicate the protective effect of turpentine oil on squalene emulsions; however, the mechanism of this phenomenon is interesting and requires additional research.

**Figure 1:**
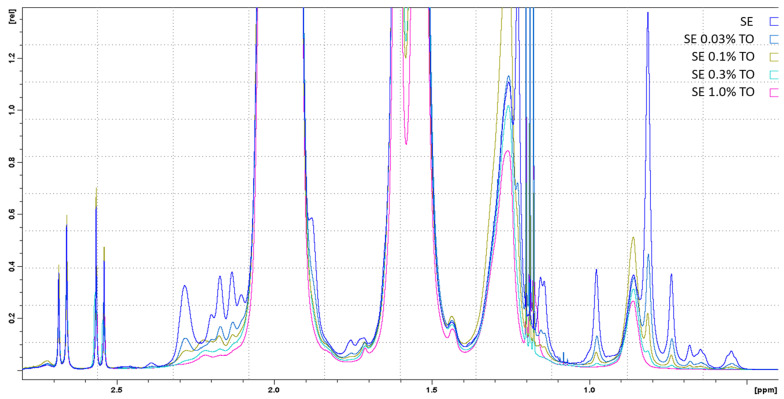
1D-1H-NMR spectra of freshly prepared emulsions with different turpentine oil contents, approximated in the terpenoid spectral region. The spectra of the emulsions are indicated by different colors and are marked in the figure in analogy with Table 1. Ethyl alcohol spectra were excluded from the analysis.

NMR spectroscopy showed that all components in the fresh (72 h after homogenization) emulsions (squalene, turpentine oil, Span^®^ 85, and TWEEN^®^ 80) were present in the spectra and were unchanged, and no additional impurities were detected in the composition of the emulsions in comparison with the original components (Figure 1). Thus, high-pressure homogenization did not affect the original components, thereby preserving their integrity and conformation. Turpentine oil did not alter the physicochemical properties of the squalene emulsion at the concentration used (0.03–1%).

**Figure 2:**
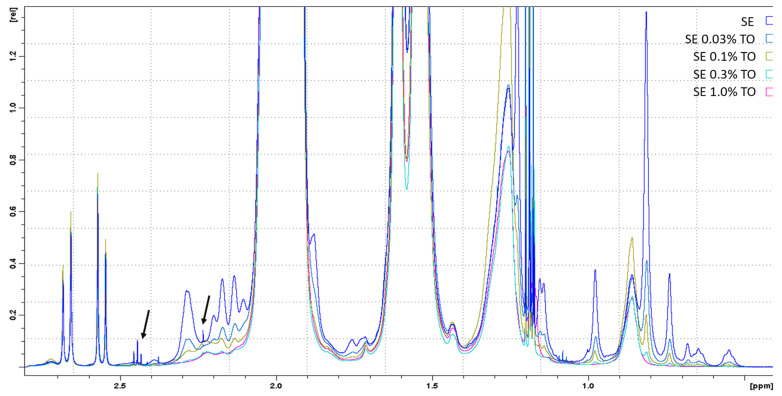
1D-1H-NMR spectra of freshly prepared emulsions with different turpentine oil contents, approximated in the terpenoid spectral region. The spectra of the emulsions are indicated with different colors and marked in the figure in analogy with Table 1. The arrows indicate minor impurities in 2-oxoglutarate (observed in the spectrum of pure squalene emulation, SE). Ethyl alcohol spectra were excluded from the analysis.

After 12 months of storage, a proportional increase in the intensity of all signals was observed with an increase in turpentine concentration. At the same time, when comparing the spectra of freshly prepared and one-year-old versions of the emulsions, changes in the spectra (minor impurities) were detected only for samples of pure squalene emulsion that did not contain turpentine oil (Figure 2, arrows).

NMR analysis showed that with an increase in the size of the micelles in the emulsion (pure squalene emulsion and emulsions with 0.03–0.1% TO) in the NMR spectrum, the intensity decreased and the width of the corresponding signals increased. Conversely, an increase in the intensity and decrease in the signals in the NMR spectra was a consequence of a decrease in the size of the micelles in the emulsions with 0.3–1.0% TO.

The physicochemical characteristics of the emulsions after 12 or 24 months of storage did not show any differences when stored at room temperature (+25 ± 2 °C) or the standard recommended temperature (+5 ± 3 °C). For all emulsions, pronounced stability indicators were revealed: the emulsions did not undergo separation and did not change in color, viscosity, pH, ζ-potential, or particle size (Table 3).

It should be noted that the emulsion samples after 24 months of storage when di-luted with deionized water (1:1 *w*/*w*) also remained highly stable and were not stratified, while the ζ-potential for samples of pure squalene emulsion was −29.8 ± 0.94 mV, while for samples of emulsion with 1% turpentine oil, the ζ-potential was −37.5 ± 1.94 mV, which generally indicates an increase in the stability properties of [69,71] squalene emulsion with the addition of turpentine oil.

**Figure 3:**
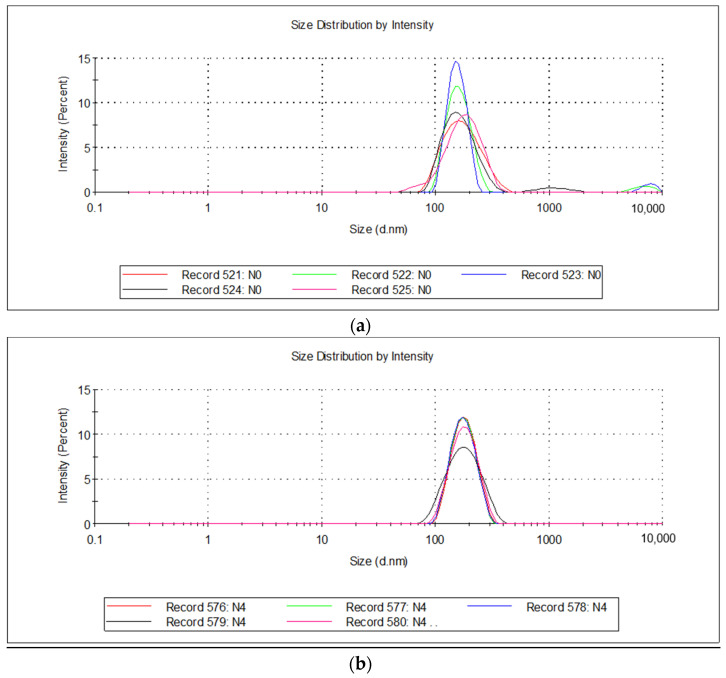
Particle size distribution of (**a**) pure squalene emulsion and (**b**) squalene emulsion with 1% turpentine oil after 24 months of storage.

These data were also confirmed by measuring the particle size in the emulsion, which showed that a large fraction of particles appeared after 24 months of storage in the pure squalene emulsion (Figure 3a), but this was not observed for the squalene emulsion with 1% turpentine oil (Figure 3b).

Thus, it was found that after long-term storage for 12 calendar months, all the studied emulsions remained stable and did not undergo separation, even at a temperature of 25 ± 2 °C. Additionally, it was revealed that the stability of the dimensional characteristics of the emulsions during storage increased and depended on the concentration of terpenitine oil.

### 3.2. Results of Safety Studies of Emulsions

**Figure 4:**
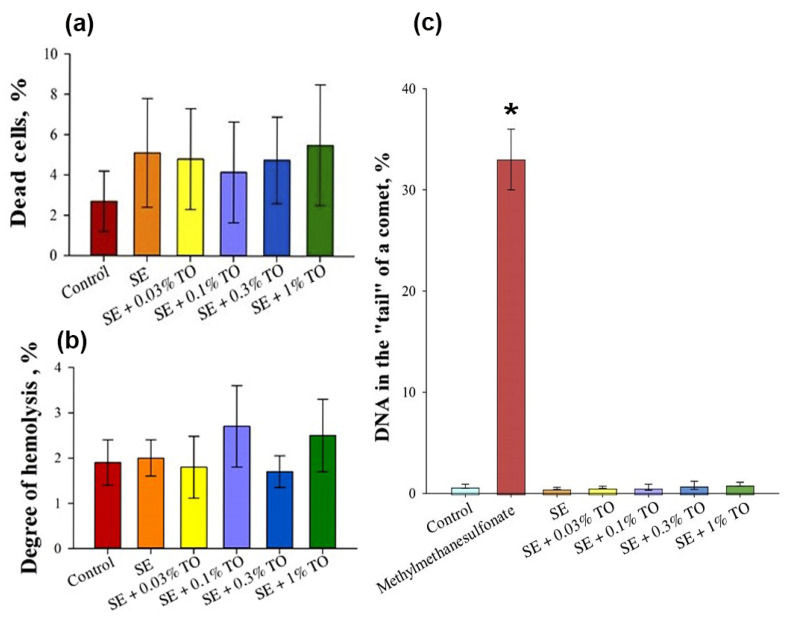
The results of the evaluation of (**a**) cytotoxic, (**b**) hemolytic, and (**c**) genotoxic effects of the emulsions in vitro (X ± SD): (**a**) cytotoxic effect of the emulsions on NIH/3T3 cells; (**b**) hemolytic activity of the emulsions during co-incubation with human peripheral blood erythrocytes; (**c**) the effect of the emulsions on the induction of DNA damage in NIH/3T3 cells. * significantly different from the control, *p* < 0.05.

Biological in vitro studies of the developed emulsions primarily aimed to assess the harmlessness of emulsions with different turpentine oil contents in in vitro test systems. It was found that the number of dead cells of mouse NIH3/T3 embryonic fibroblasts after incubation for 24 h with emulsions did not exceed 5.5 ± 3.0% of the total number of cells, which was unreliably different from the control value of 2.7 ± 1.5% (Figure 4a). Thus, the emulsions, regardless of the turpentine content, did not have a cytotoxic effect on mouse embryonic fibroblasts.

The evaluation of the hemolytic effect showed that the percentage of hemolysis during the activation of erythrocytes with emulsions with 0.03%, 0.1%, 0.3% and 1.0% turpentine oil was 1.8 ± 0.7%, 2.7 ± 0.9%, 1.7 ± 0.4% and 2.5 ± 0.8%, respectively, which did not differ from the percentage of hemolysis in the control samples with the addition of an isotonic solution and the pure squalene emulsion (Figure 4b). Thus, the emulsion with the analyzed concentrations of turpentine oil did not exhibit hemolytic activity against human peripheral blood erythrocytes.

The evaluation of the genotoxic effect showed that after incubation of the NIH/3T3 cells for 4 h with emulsion samples, the average amount of DNA in the “tail” of the comet was 0.7 ± 0.5% for all emulsions, which did not differ significantly from the control conditions, where the amount of DNA in the “tail” of the comet was 1.4 ± 0.4% (Figure 4c), and significantly differed from the amount of DNA in the “tail” of the comet after incubation of the NIH/3T3 cells for 4 h with 20 μg/mL of methyl methanesulfonate, which was 32 ± 3%. The obtained data allowed us to conclude that the emulsion had no effect on the induction of DNA damage in the NIH/3T3 cells in vitro.

An assessment of the acute toxicity of the emulsion via subcutaneous administration of the maximum injectable dose (MID) did not show a negative effect on animals. During the introduction of the emulsion, as well as throughout the study period, there were no clinical signs of abnormalities in the state of animal health (Table 4). On the third day, all animals were removed from the experiment via humane euthanasia (carbon dioxide protocol) followed by necropsy.

During the macroscopic examination of the external condition of the body; the internal surfaces and passages; the cranial cavity; thoracic, abdominal, and pelvic cavities, with their organs and tissues located in them; the neck with its organs and tissues; the skeleton; and the musculoskeletal system, no signs of the negative influence of emulsion MID were detected.

Thus, the assessment of acute toxicity of the emulsion with the addition of 1% turpentine oil by introducing a maximum injectable dose of 50 mL/kg showed a significant absence of a negative effect of the sample on the studied animal parameters; that is, the test substance with fractional daily subcutaneous administration to ICR (CD-1) mice was safe.

### 3.3. Results of Emulsion Effectiveness Assessment

The absence of immunological toxicity is one of the main requirements for medicines. The aim of this immunological study was to determine the effect of the developed emulsion on the main factors of humoral and cellular immunity, depending on the concentration of turpentine oil in the emulsions.

**Figure 5:**
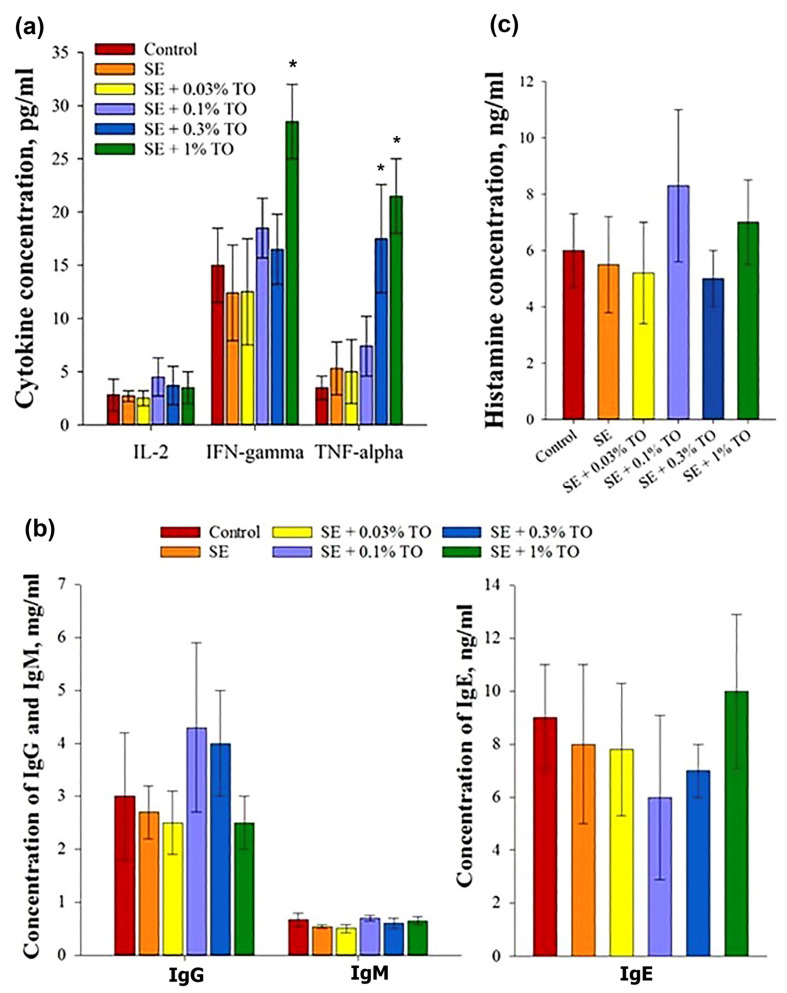
Evaluation of the effect of emulsions on the production of the main factors of humoral immunity after parenteral administration to mice (X ± SD): (**a**) production of the main cytokines, (**b**) production of immunoglobulins, (**c**) production of histamine, * significantly different from the control, *p* < 0.05.

All emulsions, regardless of the concentration of turpentine, did not change the concentration of IL-2 in the serum of mice, whereas the injection of 0.3% turpentine emulsion significantly (*p* < 0.05) increased the level of TNF-α in the serum, and the introduction of 1% of the emulsion increased the levels of TNF-α and INF-γ. The results indicated a dose-dependent increase in the production of the main cytokines studied in the experimental animals (Figure 5a). Thus, it can be confidently assumed that squalene emulsions with a turpentine oil content of 0.3–1.0% may have a safe stimulating effect on the immune system.

At the same time, there were no changes in the IgM, IgG, or IgE levels in the blood serum of mice after the introduction of the emulsion samples. The IgG content in serum after the administration of the emulsion samples did not significantly differ for different concentrations of turpentine oil, with an average value of 3.4 ± 0.9 mg/mL, and did not significantly differ from the IgG content in the blood serum samples of the control animals, which was 3 ± 1.2 mg/mL. Similar results were obtained for IgM products: the average value was 0.62 ± 0.07, which did not differ from the indicators in the control group, which was 0.67 ± 0.13 mg/mL. Like IgG and IgM products, the level of IgE in the blood serum of mice injected with emulsion samples with different turpentine contents did not significantly differ between the groups, and the average concentration of IgE in serum after administration was 7.8 ± 1.7 ng/mL, with a control value of 9 ± 2.0 ng/mL. Thus, turpentine emulsion samples at the analyzed concentrations did not affect the production of serum IgG, IgM, and IgE 24 h after administration (Figure 5b); therefore, none of the studied emulsions had their own immunogenicity.

Another criterion for activation of the immune system is the production of histamine, which is part of the granules of mast cells and is responsible for the development of inflammation. After the introduction of the emulsions, the histamine concentration did not differ significantly between the animal groups, and the average was 6.5 ± 1.5 ng/mL, with a control value of 6 ± 1.3 ng/mL. These data allowed us to conclude that the emulsion had no effect on the histamine content in the blood serum of mice, regardless of the concentration of turpentine oil (Figure 5c).

**Figure 6:**
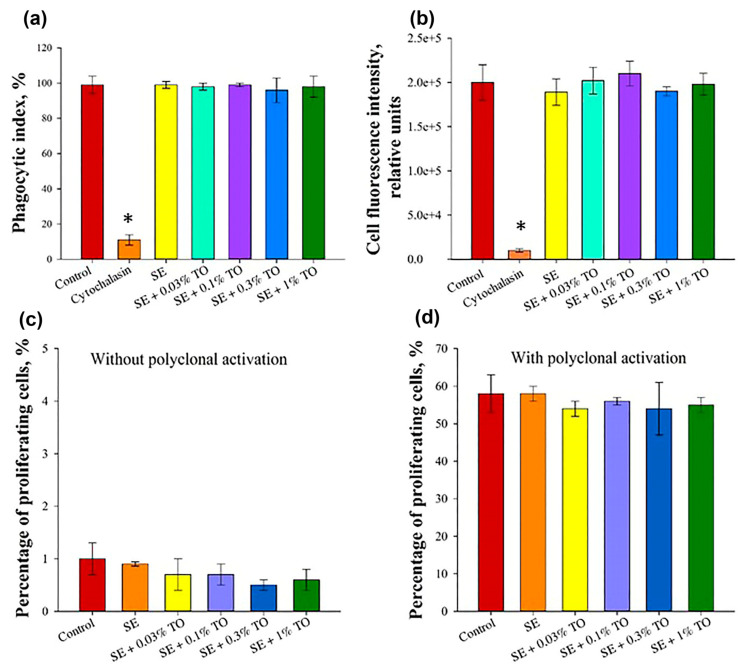
Assessment of the effect of terpenoid-based nanoemulsion on the main factors of cellular immunity (X ± SD): (**a**) phagocytic index of monocytes, (**b**) phagocytic number of monocytes, (**c**) proliferative activity of lymphocytes without polyclonal activation, (**d**) proliferative activity of lymphocytes after polyclonal activation, * significant difference from control, *p* < 0.05.

Next, we investigated the effect of the emulsions on the phagocytic activity of mouse monocytes. The average phagocytic index and phagocytic number for cells incubated with emulsion were 97.2 ± 0.8% and 197,800 ± 8700, respectively, which did not significantly differ from the control conditions (*p* ≥ 0.05), where the phagocytic index was 97 ± 3%, and the phagocytic number was 200,000 ± 20,000. At the same time, when monocytes were incubated for 30 min with 10 μg/mL of cytochalazine D, the phagocytic index and phagocytic number were 11 ± 3% and 10,000 ± 2000, respectively, which significantly differed from the control values and indicators of all the studied emulsions (Figure 6a,b).

Thus, according to the results of the studies performed, none of the emulsions with different concentrations of turpentine oil had a negative effect on the phagocytic activity of mouse peripheral blood monocytes.

When studying the effect of emulsions on mitogen-induced proliferation of peripheral blood mononuclear cells in mice, it was found that the number of Ki-67-positive cells without the addition of mitogens was less than 1.0% of the total population for all analyzed samples, which did not significantly differ from the control conditions (*p* ≥ 0.05), and the number of Ki-67-positive cells without the addition of mitogens was less than 1.0% of the total population for all analyzed samples, which did not significantly differ from the control conditions (*p* ≥ 0.05), where the number of Ki-67-positive cells that were positive cells accounted for 1.0 ± 0.3% of the total population. Upon induction of polyclonal activation, this indicator did not significantly differ between the groups and was equal to 55 ± 2% of the total population, which did not differ significantly (*p* ≥ 0.05) from the indicator of control conditions (58 ± 5%). Thus, it is shown that the emulsions with different concentrations do not have a negative effect on the proliferative activity of lymphocytes (Figure 6c,d).

**Figure 7:**
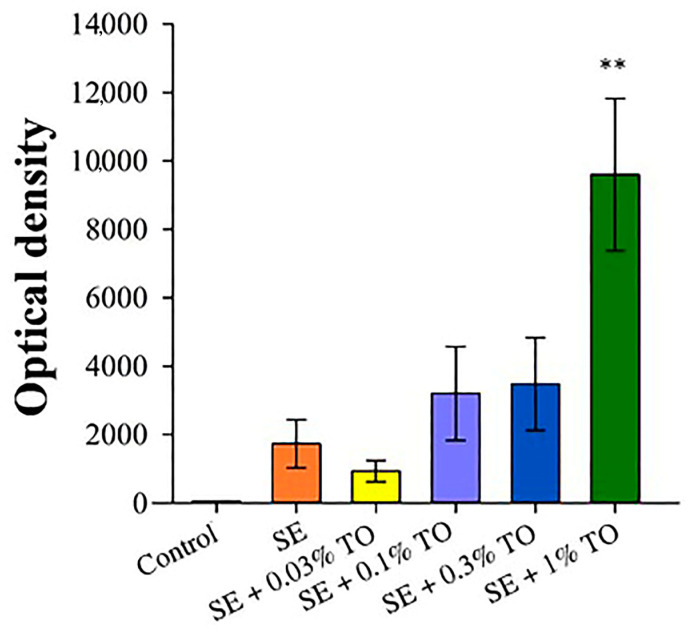
The results of measuring the titers of antigen-specific xenoantibodies in the blood serum of mice 21 days after immunization (X ± SD), **—a significant difference from the control, *p* < 0.01.

To assess the adjuvant activity of the developed emulsion, an enzyme immunoassay of mouse serum was performed 21 days after immunization with a model antigen (HSA). The highest antibody production was observed after the introduction of the squalene emulsion with 1.0% turpentine oil, which was four times higher than that of the pure squalene emulsion, similar to the generally recognized adjuvant MF59. At the same time, the remaining samples had less pronounced antibody-stimulating activity, but still significantly and many times higher (*p* < 0.01) than the values of the control group (Figure 7), which indicates pronounced immunostimulatory activity of the studied emulsions and a direct dependence of the level of this activity on the concentration of turpentine oil.

Thus, it can be concluded that the studied versions of the emulsion have pronounced immunostimulatory activity, and the identified effects are dose-dependent on the turpentine oil concentration.

## 4. Discussion

The need to develop technologies for the preparation of stable homogeneous ultra-dispersed emulsions is due to the increasing demand in medicine, pharmacology, and veterinary medicine. Based on such dispersed systems, adjuvants and other immunostimulatory emulsions for local or general exposure to medicinal substances and biologically active compounds have been intensively developed. It is expected that the development of a wide range of drugs on a dispersed basis and the use of various nanocontainers for targeted delivery of drugs to various organs and tissues can provide new methods of treatment, as well as a much more pronounced therapeutic effect of known drugs with a reduction in active substance doses. In turn, a replacement for shark squalene with equivalent or better vaccine adjuvant properties when appropriately formulated but sourced sustainably would be desirable to alleviate the population pressure on sharks and to secure the long-term future of this class of vaccine adjuvants [72]. We believe that the natural terpenoids in turpentine oil are promising alternatives.

Summarizing the results of this study, it can be concluded that the obtained emulsions with different concentrations of turpentine oil exhibited a dose-dependent effect, depending on the concentration of turpentine oil, both in terms of efficiency and storage stability. The stability of squalene emulsions and their potential as adjuvants has long been considered [73]. Owing to the presence of six unsaturated bonds in the squalene structure, it can undergo oxidation. Hydroperoxides, superoxide anions, singlet acids, hydroxyl radicals, and other compounds can act as oxidizing agents. Thus, in the presence of synthetic oxygen, the accumulation of squalene peroxides was observed during the oxidation of squalene [74], which was further degraded to the corresponding carbonyl and carboxyl compounds. A similar process was observed during the oxidation of squalene by ozone [75]. The resulting products of squalene oxidation should stabilize the emulsion during storage, acting as surfactants and complementing the function of Tween 80 in this emulsion, which logically agrees with the data on the increase in the peak of Tween 80 in the NMR spectra after 12 months of storage (Figure 1 and Figure 2). It is assumed that, in the same way, turpentine oil, consisting mainly of terpenes of various structures, when introduced into a squalene emulsion can lead to an analogous effect and further stabilize the structure of the emulsion particles, which will be further investigated.

It is important to note that all studied emulsions showed a high safety profile regardless of the concentration of used turpentine oil. The NMR method additionally revealed that the studied versions of emulsions do not interact with serum proteins without causing adsorption on the surface of protein particles; that is, the resulting emulsion can be stored both separately and together with vaccine antigens without changing their antigenic properties [76,77]. At the same time, it is the emulation variant with the highest concentration of turpentine oil (1%) that has the most pronounced adjuvant activity and the most pronounced stability indicators when stored at room temperature. Although none of the studied versions of emulsions changed the content of IL-2 and did not have their own immunogenicity, a significant increase in the levels of interferon-γ and TNF-α in the blood serum of animals after the introduction of an emulsion with 1% turpentine oil indicated that the resulting emulsion may have a pleiotropic effect that stimulates the production of major cytokines and may have a pronounced immuno-stimulating effect.

## 5. Conclusions

It can be concluded that our proposed method of producing squalene emulsions with 1% turpentine oil using high-pressure homogenization is effective and provides a stable, monomodal, and reliably safe ultra-dispersed emulsion with pronounced immunopotentiating properties.

## Figures and Tables

**Table 1:** Results of the evaluation of the physico-chemical characteristics of the emulsions after preparation.

Emulsion	% Water/Oil	PH	Dynamic Viscosity, cPs	Particle Size, nm	ζ-Potential, mV
Squalene emulsion (SE)	95/5	6.01 ± 0.01	1.194 ± 0.009	175.4 ± 54.57	−3.34 ± 1.16
SE + 0.03% turpentine oil (TO)	95/5	5.99 ± 0.01	1.148 ± 0.008	181.5 ± 51.97	−3.19 ± 1.43
SE + 0.1% TO	94/6	5.97 ± 0.01	1.194 ± 0.005	169.1 ± 43.49	−3.01 ± 0.6
SE + 0.3% TO	94/6	5.98 ± 0.01	1.176 ± 0.005	174.4 ± 58.84	−3.36 ± 0.69
SE + 1.0% TO	95/5	5.98 ± 0.01	1.186 ± 0.005	178.4 ± 49.41	−2.55 ± 0.88

**Table 2:** TBARS measurement results.

Emulsion	t0_min_	t30_min_	t24_h_
SE	1.15 ± 0.14	8.28 ± 0.44	16.45 ± 2.07 ***
SE + 0.03% TO	1.34 ± 0.22	11.31 ± 0.30	12.56 ± 1.14
SE + 0.1% TO	1.82 ± 0.19	12.90 ± 0.56	15.82 ± 1.05 *
SE + 0.3% TO	1.37 ± 0.13	27.39 ± 0.80	29.06 ± 2.54
SE + 1.0% TO	1.95 ± 0.21	23.55 ± 1.10	29.13 ± 3.17 *
10% lecithin emulsion	1.28 ± 0.10	67.91 ± 2.52	78.15 ± 5.11 *

* *p* ≤ 0.05 relative to t30_min_; *** *p* ≤ 0.001 relative to t30_min_.

**Table 3:** Results of the evaluation of the physicochemical characteristics of the emulsions after 12 and 24 month of storage (+5 ± 3 °C).

Emulsion	PH	Dynamic Viscosity, cPs	Particle Size, nm		ζ-Potential, mV *	
			12 Month	24 Month	12 Month	24 Month
SE	6.00 ± 0.01	1.193 ± 0.009	169.8 ± 14.9	162.2 ± 16.1	−4.05 ± 0.42	−5.46 ± 0.75
SE + 0.03% TO	5.99 ± 0.01	1.147 ± 0.009	175.3 ± 16.1	172.0 ± 5.9	−4.82 ± 0.66	−7.23 ± 0.54
SE + 0.1% TO	5.98 ± 0.01	1.196 ± 0.007	154.5 ± 10.4	159.3 ± 10.4	−4.18 ± 0.52	−6.44 ± 0.39
SE + 0.3% TO	5.97 ± 0.02	1.177 ± 0.008	174.8 ± 9.7	180.3 ± 7.8	−4.34 ± 1.08	−6.35 ± 0.78
SE + 1.0% TO	5.99 ± 0.01	1.19 ± 0.008	164.1 ± 5.9	174.8 ± 9.7	−4.1 ± 0.86	−5.18 ± 0.91

* The ζ-potential values of the emulsions were determined after dilution with citrate buffer.

**Table 4:** Results of evaluation of animal state after subcutaneous administration of MID of squalene emulsion with maximum concentration of turpentine oil.

	Control (Saline)		SE + 1% TO	
	$\bar{X}\pm SD$	*n*	$\bar{X}\pm SD$	*n*
Day 1	30.4 ± 0.6	3	30.0 ± 1.3	8
Day 2	30.3 ± 0.8	3	29.9 ± 1.2	8
Day 3	30.4 ± 0.4	3	30.0 ± 0.9	8
Day 2	−0.2 ± 0.8	3	−0.2 ± 0.7	8
Day 3	0.0 ± 0.6	3	0.2 ± 1.3	8

## Data Availability

The data presented in this study are contained within this article.
